# The New York State Academy of Veterinary Science and Comparative Pathology

**Published:** 1886-07

**Authors:** 


### THE NEW YORK STATE ACADEMY OF VETERINARY SCIENCE AND COMPARATIVE PATHOLOGY.

During the past season the Academy has made very good progress in membership, in the choice of which it is very select.

No meeting of the Academy passes without interesting matter for debate, and subjects are brought up from time to time, which are not only interesting to every member, but to the public as well.

In this respect, questions are often asked by those not members, which give rise to useful and important discussion. Recectly, Mr. Henry Bergh, President of the American Society for prevention of cruelty to Animals, sent in the transcript of a letter, which he had previously sent to the *press*, on the question of "Hot Shoeing for Horses," or applying the shoe hot to the horse's foot while fitting.

The President then introduced Dr. Robert Ward, F. R. C. V. S., State Veterinarian of Baltimore, Md., who addressed the meeting with reference to the Code of Ethics relating to Veterinary Medicine, contrasting what is in existence with what ought to be; expressing his views by stating that he was ever willing to accept or recognize any graduate of any legitimate school who was a gentleman. The speaker said unfortunately that there were graduates from some schools who were not always gentlemen.

The doctor then entered on the subject of "State Medicine," or *Preventative Medicine*, which he said hardly, or rather did not exist in this country, and asked whether any member present had ever heard of the Veterinarian having been called upon by the State to prevent the breaking out of contagious diseases?

This was of the utmost importance to the State, and the prevention of contagious diseases was where the higher duties of the Veterinarian were most needed by the community at large, because he had not the slightest doubt but that many thousands of human beings die annually from using the milk and flesh of diseased animals. And while we hear very often of Boards of Health enforcing laws against the spread of disease, and the "stamping out" process, yet we never hear one word about the prevention of disease, while here is the field for the scientific work of the Veterinarian and Physician. And the State should see to it that such work should be entered upon at once, as the best, and in fact the only means to preserve the State from a possible future loss due to animal plagues.

The doctor then described in detail the different contagious diseases to which live stock are subject, particularly such as become epizootic in character, viz.: Contagious Pleuro-Pneumonia, Tuberculosis, Anthrax, Hog Cholera, and even Chicken Cholera. Glanders also came in for a share of comment. He quoted several authors, particularly the great sanitarian Fleming, and the able Professor Walley, on the system of inoculation to prevent the outbreak of contagious diseases, especially Pleuro-Pneumonia in cattle, and paid tribute to Pasteur in his investigations.

The doctor was of the opinion that inoculation might be carried considerably further.

A difference of opinion does exist in regard to the use of the milk and flesh of animals affected with contagious Pleuro-Pneumonia, some authorities claiming it to be harmless, while that of an animal affected with Tuberculosis is admitted by all to be dangerous, it being comunicable to mankind.

Dr. Ward went still further, and stated that the "flesh of any animal affected with any disease in which the blood was improperly decarbonized, was unfit for human food."

During the discourse he exhibited some elegant colored drawings of Pathological specimens of the blood, lungs, kidneys, etc., etc., of animals suffering from the afore-mentioned diseases, many of the drawings were original, and others were from slides taken from the laboratory of the "Johns Hopkins University," Md. Beautiful specimens of the Bacilli and Microbes peculiar to contagious diseases were plainly presented, but in some cases the question arose whether they were the cause or result of the disease.

Dr. Ward's paper called forth considerable discussion.

Dr. Gotthiel said, an animal showing symptoms of any disease, whether before being slaughtered, or after, was not fit for human food, and should be condemned by the inspectors; this should be made compulsory by law. In this he was supported by nearly every member present.

Dr. Earl warned the Academy to be very careful about advocating *law* on this subject, as statutes to this effect would cut off a vast supply of flesh, which, even under suspicious circumstances, made very good food, when *properly cooked.*

Dr. Hamill said that was a question for the political economists. As a scientific body the Academy could only take up the question "Was diseased animal flesh fit for human food?" and while agreeing with Dr. Earl, in regard to the evil of lessening the supply, he believed it was not fit.

Drs. Gottheil, Heard and Meyer sifted through many authorties on this point, showing that there are stages in most all diseases, when there was little or no danger to be apprehened, but the above gentleman claimed better *no* flesh meat than diseased meat.

The following resolution was unanimously adopted:

RESOLVED, that this Academy advocate the passage of proper laws for the establishment of public Abattoirs, where, under the observation of *properly qualified inspectors*, all animals to be used for human food, must be slaughtered.

And further, that all animals found suffering from anthrax, tuberculosis, actinomycosis, trichinoses, hog-cholera pleuropneumonia, and chicken-cholera, should be *entirely excluded* as unfit for consumption.

It was then moved and seconded that a vote of thanks and the support of the Academy be tendered to Dr. Ward, in his noble efforts to elevate the Veterinary profession in America.

